# Signature of weak-antilocalization in sputtered topological insulator Bi_2_Se_3_ thin films with varying thickness

**DOI:** 10.1038/s41598-022-13600-8

**Published:** 2022-06-13

**Authors:** Sudhanshu Gautam, V. Aggarwal, Bheem Singh, V. P. S. Awana, Ramakrishnan Ganesan, S. S. Kushvaha

**Affiliations:** 1 CSIR- National Physical Laboratory, Dr. K.S. Krishnan Marg, New Delhi, India 110012; 2 Academy of Scientific and Innovative Research (AcSIR), Ghaziabad, India 201002; 3 Department of Chemistry, Birla Institute of Technology and Science (BITS), Pilani, Hyderabad Campus, Jawahar Nagar, Kapra Mandal, Medchal District, Hyderabad, Telangana 500078 India

**Keywords:** Physics, Topological matter

## Abstract

We report a low-temperature magneto transport study of Bi_2_Se_3_ thin films of different thicknesses (40, 80 and 160 nm), deposited on sapphire (0001) substrates, using radio frequency magnetron sputtering technique. The high-resolution x-ray diffraction measurements revealed the growth of rhombohedral c-axis {0003n} oriented Bi_2_Se_3_ films on sapphire (0001). Vibrational modes of Bi_2_Se_3_ thin films were obtained in the low wavenumber region using Raman spectroscopy. The surface roughness of sputtered Bi_2_Se_3_ thin films on sapphire (0001) substrates were obtained to be ~ 2.26–6.45 nm. The chemical and electronic state of the deposited Bi_2_Se_3_ was confirmed by X-ray photoelectron spectroscopy and it showed the formation of Bi_2_Se_3_ compound. Resistivity versus temperature measurements show the metallic nature of Bi_2_Se_3_ films and a slight up-turn transition in resistivity at lower temperatures < 25 K. The positive magneto-resistance value of Bi_2_Se_3_ films measured at low temperatures (2–100 K) confirmed the gapless topological surface states in Bi_2_Se_3_ thin films. The quantum correction to the magnetoconductivity of thin films in low magnetic field is done by employing Hikami–Larkin–Nagaoka theory and the calculated value of coefficient ‘α’ (defining number of conduction channels) was found to be 0.65, 0.83 and 1.56 for film thickness of 40, 80 and 160 nm, respectively. These observations indicate that the top and bottom surface states are coupled with the bulk states and the conduction mechanism in Bi_2_Se_3_ thin films varied with the film thicknesses.

## Introduction

Topological insulators (TIs) have been the subject of much interest for over a decade due to their unique properties, owing to the presence of time reversal symmetry and strong spin orbit coupling, which bring about exceptional properties that have been used in various applications and devices such as spintronics, quantum computing, and quantum anomalous Hall effect^1–4^. These are a class of quantum matter with their bulk being insulating and the surface composed of the odd number of Dirac cones. TIs show exotic properties such as protection against backscattering from non-magnetic impurities as well as defects that do not alter time reversal symmetry (TRS) due to the presence of a Π Berry phase on the Fermi surface^4,5^. A plethora of magneto-transport experiments have indicated that the surface state of a TI is topologically protected by TRS i.e., they are not easily perturbed by non-magnetic disturbances or defects^6–10^. However, TRS can be suppressed in a perpendicular magnetic field, resulting in a positive magneto-resistance (MR) which is an interference phenomenon arising from two-dimensional (2D) quantum interference mechanisms in thin films and nano-devices, known as the weak antilocalization (WAL) effect^11^.

Among the topological insulators, Bi_2_Se_3_ has bandgap of ~ 0.3 eV and simple electronic band structure which consist of a single Dirac cone surface state with zero or negligible band gap in bulk form and thus emerging as an ideal material for TIs^12–14^. However, bulk of Bi_2_Se_3_ contains selenium vacancies which pull down the Fermi level into bulk conduction band imparting subtle bulk conductivity^15^. Generally, thin films offer more control over the contribution of bulk carriers towards reducing bulk conductance, and hence their fabrication has been of great interest for exploring fundamental research and practical applications. Bi_2_Se_3_ films and nanoribbons have been observed to show unsaturated linear magneto-resistance (LMR) under the influence of high field which originates from linear Dirac surface dispersion^15^. Various techniques such as thermal evaporation, pulsed laser deposition, and molecular beam epitaxial (MBE) growth have been previously employed^16,17–21^, however tailoring the thickness of film has been a great challenge. On the other hand, magnetron sputtering affords precise thickness control with uniform and high deposition rate for the preparation of large area thin films. As per existing literature, limited reports are available on Bi_2_Se_3_ thin films grown by magnetron sputtering for study of WAL behavior in sputtered film^14,22,23^. The contributions of surface states and bulk carriers to total transport are highly dependent on the thickness of the film^16^. The bulk states of Bi_2_Se_3_ are generally found to be conducting because of some inherent defects or natural doping, which causes a coupling effect between top and bottom surfaces states^16^. The WAL effect observed under low magnetic fields is found to be suppressed in thicker films because the surface effect is obstructed by the major bulk conductance of film due to the less surface to volume ratio in thick films. The cusp like magnetoconductance data at lower fields has been fitted with Hikami–Larkin–Nagaoka (HLN) quantum interference model to determine the number of independent conduction channels (α) in these films^15–20^. In literature, it is given that the value of α is equal to 0.5 for single coherent transport channel and 1 for two coherent transport channel (top and bottom) which are completely independent of each other. There may be two conducting surface channels that are partially coupled by conducting bulk if the value of α lies between 0.5 and 1. And α greater than 1 shows the complete decoupling of transport channel^11,14,22^. In addition to WAL effect, a linear MR effect was also seen in many TI systems which also contribute to the total conductance of the system and can be also fitted by modified HLN equation^24^.

In this article, we have studied the thickness (40–160 nm) dependent low temperature transport properties of sputtered Bi_2_Se_3_ thin films deposited on sapphire (0001) substrates. Low temperature transport measurements were performed up to 2 K in a varying magnetic field (− 12 to 12 Tesla). The WAL effect is observed at low temperatures and a WAL cusp is found in the low magnetic field region for films with a larger resistivity (40 and 80 nm) and exhibit the strong WAL effect. The active number of transport channels ‘α’ was estimated by applying HLN/modified HLN theory which found to be increased from 0.65 to 1.56 with increasing Bi_2_Se_3_ film thickness from 40 to 160 nm on sapphire (0001) substrates.

## Result and discussion

### Structural and electronic properties of Bi_2_Se_3_ thin films on sapphire (0001)

**Figure 1:**
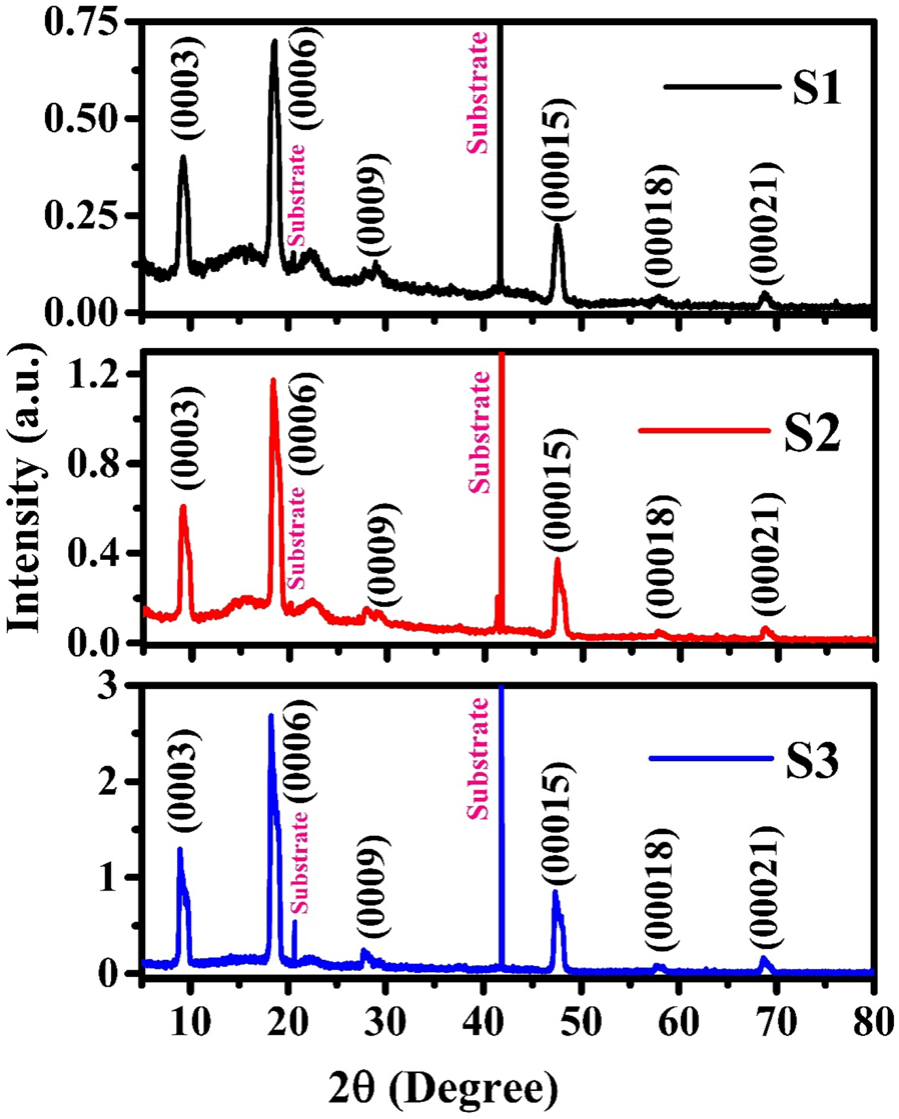
HR-XRD 2θ-ɷ scans of Bi_2_Se_3_ thin films on sapphire (0001) of samples S1 (40 nm), S2 (80 nm) and S3(160 nm).

Figure 1 shows the high-resolution x-ray diffraction (HR-XRD) 2θ-ω scan of Bi_2_Se_3_ films on sapphire (0001) with thicknesses of 40 (S1), 80 (S2) and 160 nm (S3). HR-XRD pattern clearly shows the rhombohedral structure of Bi_2_Se_3_ and all peaks are belonging to the {003n} family of planes, which indicate the highly c-axis oriented growth of Bi_2_Se_3_ thin films on sapphire (0001) substrates. The diffraction peaks of sapphire (0001) substrate were found at 2θ values of 21 and 41°. High intense and sharp peak is observed for Bi_2_Se_3_ (0006) plane indicates the good crystalline quality of Bi_2_Se_3_ films^20^. Bi_2_Se_3_ (0006) peak become intense with increasing film thickness from 40 nm (S1) to 160 nm (S3).

**Figure 2:**
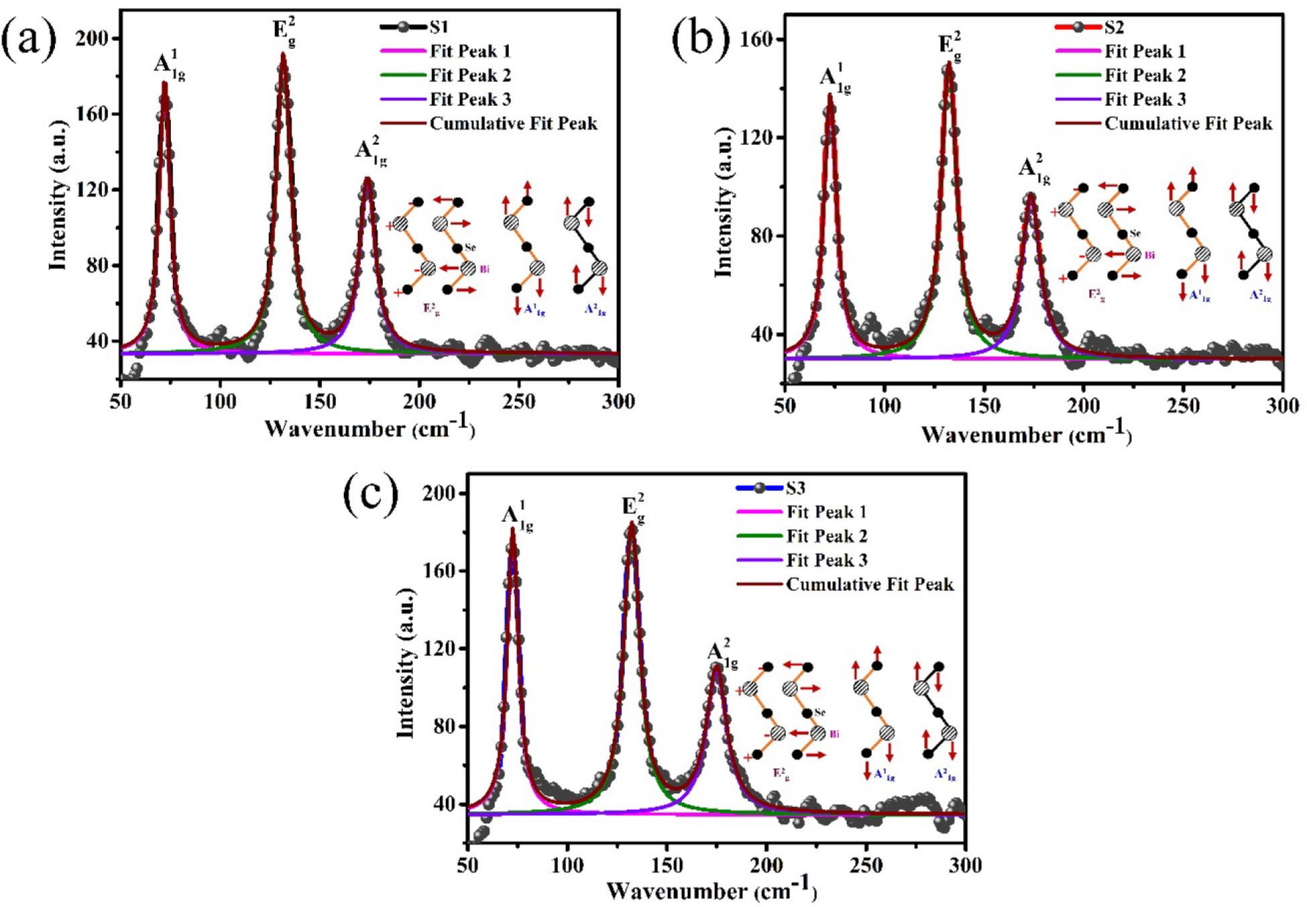
Raman vibrational modes of Bi_2_Se_3_ thin films on sapphire (0001) for samples: (**a**) S1, (**b**) S2 and (**c**) S3.

Figure 2a–c show the Lorentzian fitted Raman plot of Bi_2_Se_3_ thin films on sapphire (0001) for samples S1, S2 and S3, respectively. In the Raman spectra, three Raman peaks namely A^1^_1g_, E^2^_g_, and A^2^_1g_ were detected in low wavenumber region for all samples (S1-S3). The atomic vibrations corresponding to each Raman mode is shown along with Lorentzian fitted Raman plots. The peak positions of A^1^_1g_, E^2^_g_, and A^2^_1g_ modes and their corresponding full width at half maximum (FWHM) are tabulated in Table 1. The FWHM values for these prominent vibrational modes for sample S1 is low and comparable to reported literature, that revealed the good structural quality of sputtered Bi_2_Se_3_ thin film^26,27^.

**Table 1:** Room temperature Raman data for Bi_2_Se_3_ thin films on sapphire (0001) [S1-S3].

Sample name	A^1^_1g_		E^2^_g_		A^2^_1g_	
	Peak position (cm^−1^)	FWHM (c m^−1^)	Peak position (cm^−1^)	FWHM (cm^−1^)	Peak position (cm^−1^)	FWHM (cm^−1^)
S1	72.11	6.43	131.73	8.71	173.87	9.59
S2	72.75	7.49	132.31	9.38	173.52	11.02
S3	72.64	6.78	132.33	9.69	174.92	12.46

**Figure 3:**
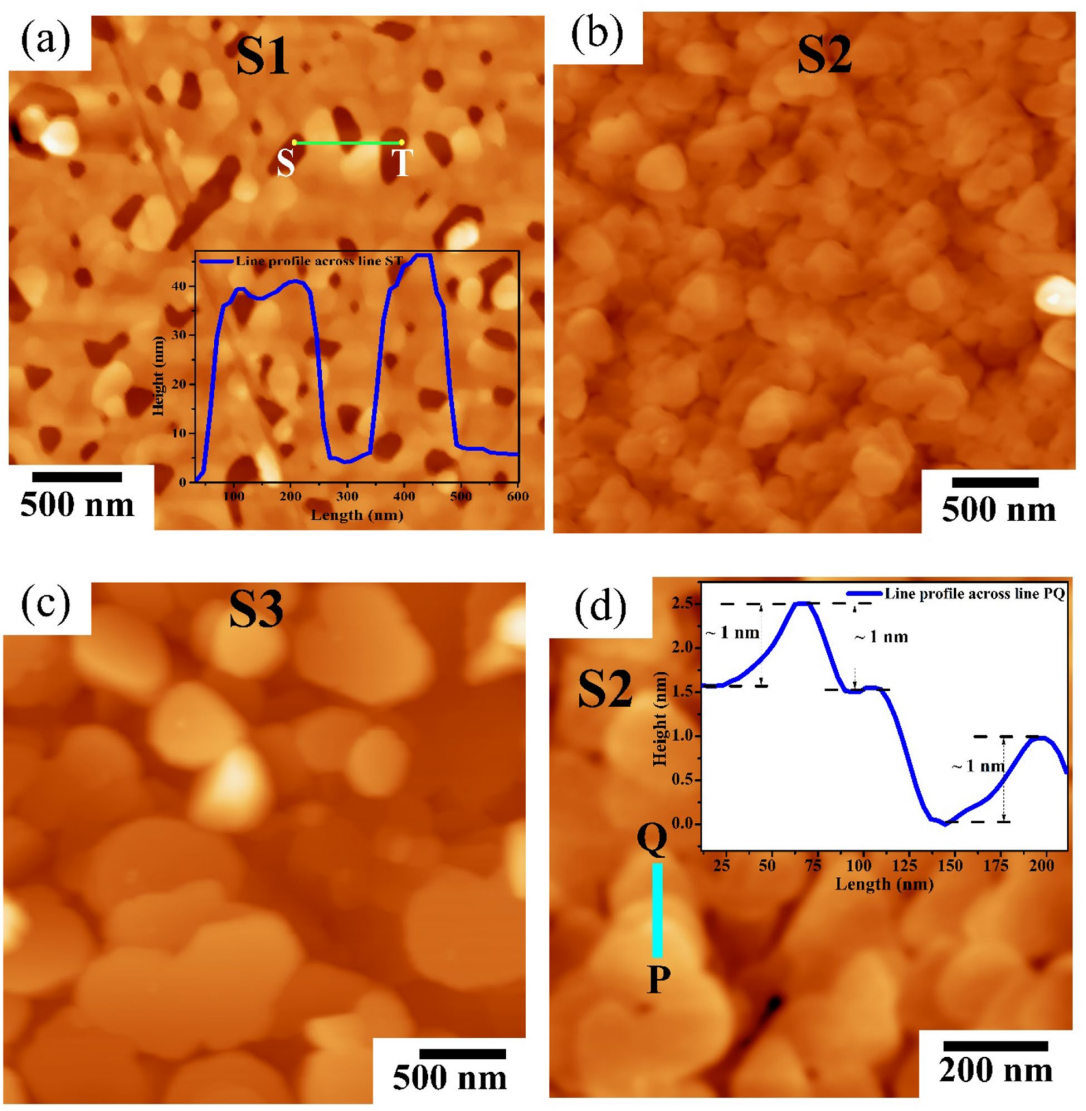
Large scan area (3 μm)^2^ topographic tapping mode AFM images for samples S1 (**a**), S2 (**b**) and S3 (**c**). (**d**) AFM image of sample S2 with scan area of (1 μm)^2^ and inset shows the line profile across PQ line. Inset of (**a**) shows the line profile along ST line.

The surface morphology of samples S1-S3 were characterized using atomic force microscopy (AFM) and AFM images are shown in Fig. 3a–c. Sample S1 shows the inter-connected truncated triangular and hexagonal shape grains-like surface with some triangular or hexagonal pits (Fig. 3a) and the surface coverage is nearly ~ 90%. The root mean square (rms) surface roughness for sample S1 was obtained to be 5.85 nm for scan area (3 µm)^2^. The inset of Fig. 3a displays the line profile of sample S1 along line ST and it also showed the height of Bi_2_Se_3_ thin films falls ~ 40 nm. We can see in Fig. 3b that with the increase in film thickness, the surface coverage in sample S2 increases to ~ 100%, and rms surface roughness decreases to 2.26 nm. For thicker sample S3, the large coalesce layers of Bi_2_Se_3_ thin films can be seen with few random shape islands as shown in Fig. 3c and the rms surface roughness for sample S3 is 6.45 nm for scan area (3 µm)^2^. The high magnification AFM image of sample S2 in Fig. 3d clearly shows the formation of layered Bi_2_Se_3_ thin films_._ The line profile across the PQ line in Fig. 3d showed the step height of ~ 1 nm (inset of Fig. 3d) and it is similar to the thickness of one quintuple layer of Bi_2_Se_3_ (0.96 nm)^28^.

**Figure 4:**
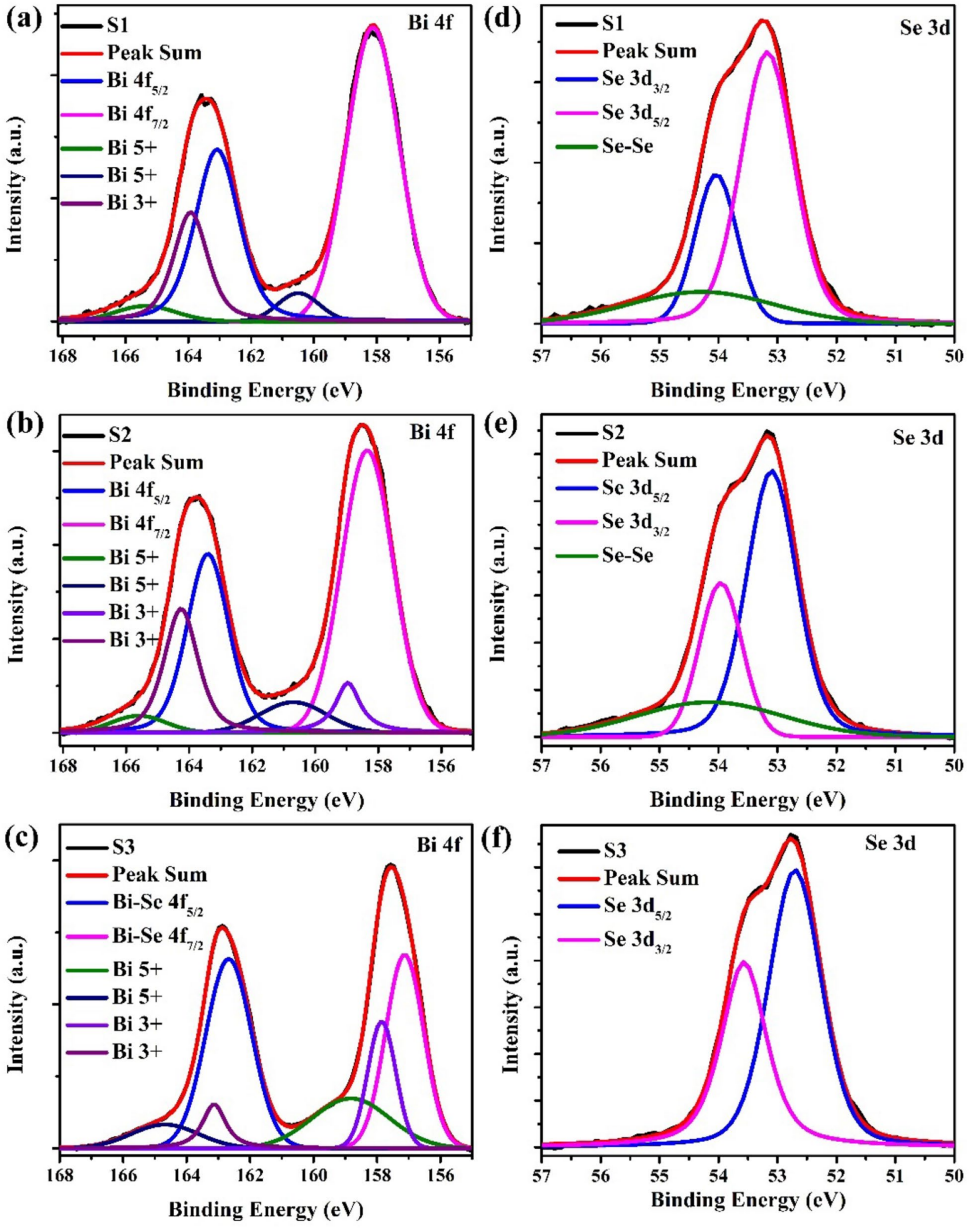
XPS spectra of Bi_2_Se_3_ thin films: Bi 4f core level spectra in the binding energy range from 155 to 168 eV for samples S1 (**a**), S2 (**b**) and S3 (**c**). Se 3d core level spectra in the binding energy range of 50 to 57 eV for samples: (**d**) S1, (**e**) S2 and (**f**) S3.

To study the chemical and electronic states of magnetron sputtering deposited Bi_2_Se_3_ thin films on sapphire (0001), we have performed the ex-situ x-ray photoelectron spectroscopy (XPS) measurements. Figure 4a–c shows the core level XPS scan of Bi 4f of all samples (S1-S3) in the binding energy range of 155–168 eV.

The two-measure peaks centered at positions (163.08, 158.06), (163.43, 158.36), and (162.67, 157.13) eV represents the two spin–orbit coupled 4f components i.e., (4f_5/2_, 4f_7/2_), for samples S1, S2 and S3, respectively^20,29–31^. The ~ 5 eV binding energy difference in these two spin–orbit coupled peaks confirms the formation of Bi_2_Se_3_ for all the samples^26^. These two peaks are fitted using the Lorentzian-Gaussian combined function. The 4f_5/2_ and 4f_7/2_ peaks are deconvoluted into three peaks which correspond to Bi 5 + , Bi 3 + , and Bi 3 + (Bi-Se) oxidation state. The peak positions of all the oxidation states of Bi 4f are given in Table 2.

**Table 2:** Fitted XPS peak positions of Bi-4f core level spectra Se-3d core level spectra for samples S1–S3.

Sample index	Bi-4f peak positions (eV)						Se-3d peak position (eV)		
	4f_5/2_			4f_7/2_			Se-Bi (3d_3/2_)	Se-Bi (3d_7/2_)	Se-Se
	Bi-O (+ 5)	Bi-O (+ 3)	Bi-Se	Bi-O (+ 5)	Bi-O (+ 3)	Bi-Se			
S1	165.48	163.97	163.08	160.46	–-	158.06	54.02	53.18	54.40
S2	165.77	164.29	163.43	160.74	158.99	158.36	53.96	53.07	54.36
S3	164.83	163.15	162.67	158.92	157.86	157.13	53.57	52.68	–

Here, the Bi 5 + and Bi 3 + peaks, on the higher binding energy side of the Bi-Se 4f peak, shows some trace of Bi-O bonds along with Bi_2_Se_3_ and it may occur due to oxidation of Bi_2_Se_3_ to some extent due to postdeposition air exposure^29^. Also, in all three samples, the Bi 4f_5/2_ as well 4f_7/2_ peaks are found to be blue-shifted as compared to those reported by Nascimento et al., who found the Bi 4f_5/2_ and 4f_7/2_ peaks at 161.9 and 156.6 eV, respectively for elemental Bi^30^. In addition, core level scans for Se 3d have been taken for these samples in the binding energy range of 50–57 eV as shown in Fig. 4d–f. The single broad and highly asymmetric Se 3d peaks in all the samples are deconvoluted into two new peaks corresponding to Se 3d_3/2_ and 3d_5/2_ using Lorentzian-Gaussian fitted function and their peak positions are given in Table 2^26,29–31^. In samples S1 and S2, an additional broad peak was observed on the higher binding energy side of Se 3d_3/2_ and 3d_5/2_ peaks for the Se-Se bond^26,28^. As there is no trace of a Se-O bond found, the presence of Bi_2_Se_(3-x)_O_x_ complex formation is ruled out in these samples. These Se 3d peaks are found to be redshifted as compared with the ones reported by other groups^20^. This opposite kind of shift found in Bi 4f and Se 3d peaks confirms the formation of Bi-Se bond, where the charge is transferred from Bi to Se^26,32^. This shift is the same as observed in pure bulk Bi_2_Se_3_ single crystals which assure the formation of high-purity magnetron sputtering deposited Bi_2_Se_3_ thin film on sapphire (0001)^26^.

### Transport properties of sputtered Bi_2_Se_3_ thin films

**Figure 5:**
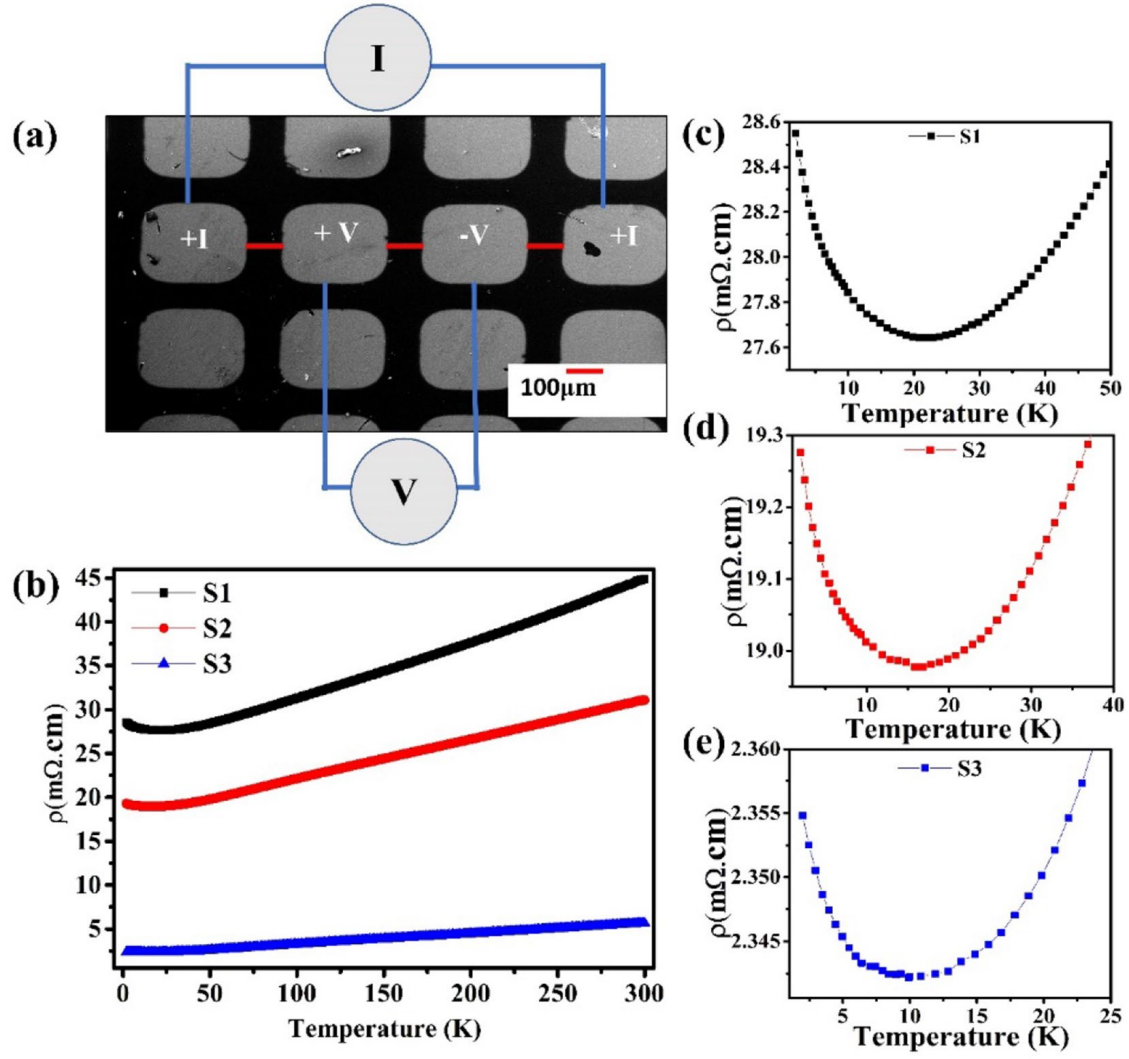
(**a**) SEM image of the fabricated device for low temperature transport measurements and (**b**) temperature dependent (2–300 K) resistivity curves for S1–S3 samples. (**c**–**e**) Represent the resistivity data at narrow temperature range of 2–50 K for S1, S2 and S3 samples, respectively.

The transport measurements on Bi_2_Se_3_ thin films were performed on device fabricated by depositing Cr/Au contacts (~ 80 nm) using mask and it can be clearly seen the formation of proper metal contact with uniform spacing as shown in scanning electron microscopy (SEM) image (Fig. 5a). Resistivity vs temperature measurements were performed and electrical resistivity (ρ) as a function of temperature (T) was plotted for all the samples (Fig. 5b) in the temperature range of 300 to 2 K. From resistivity data, it is clear that the sample S1 showed high resistivity while the sample S3 showed low metallic resistivity. Therefore, continuous decrement in resistivity is seen with increasing film thickness which suggest that the contribution of bulk states to the total conduction is increased with increasing thickness of film. On the other hand, the linear declination in resistivity with decreasing temperatures up to (~ 25 K) for all samples shows the pure metallic nature of Bi_2_Se_3_ films proposing that the total conductance at relatively higher temperatures is governed by bulk states^33^. Resistivity vs temperature curves for all the samples shows that after reaching a minimum value of resistivity, there is a smooth upturn in metallic resistivity at temperatures of 21 K, 16 K and 11 K for samples S1, S2 and S3, respectively (Fig. 5c–e). The onset of insulating behavior, beyond which the resistivity increases, reveals that thickness of films shows pronounced effect on conductivity and the transition temperature from metallic to insulating behavior is shifted towards lower temperatures as thickness increases. It is reported that in low temperature regions, surface carriers are the major source, which is responsible for conductivity because bulk carriers likely freeze at a lower temperature, while bulk carriers have dominating contributions to the conductivity in higher temperature regions^22^.

**Figure 6:**
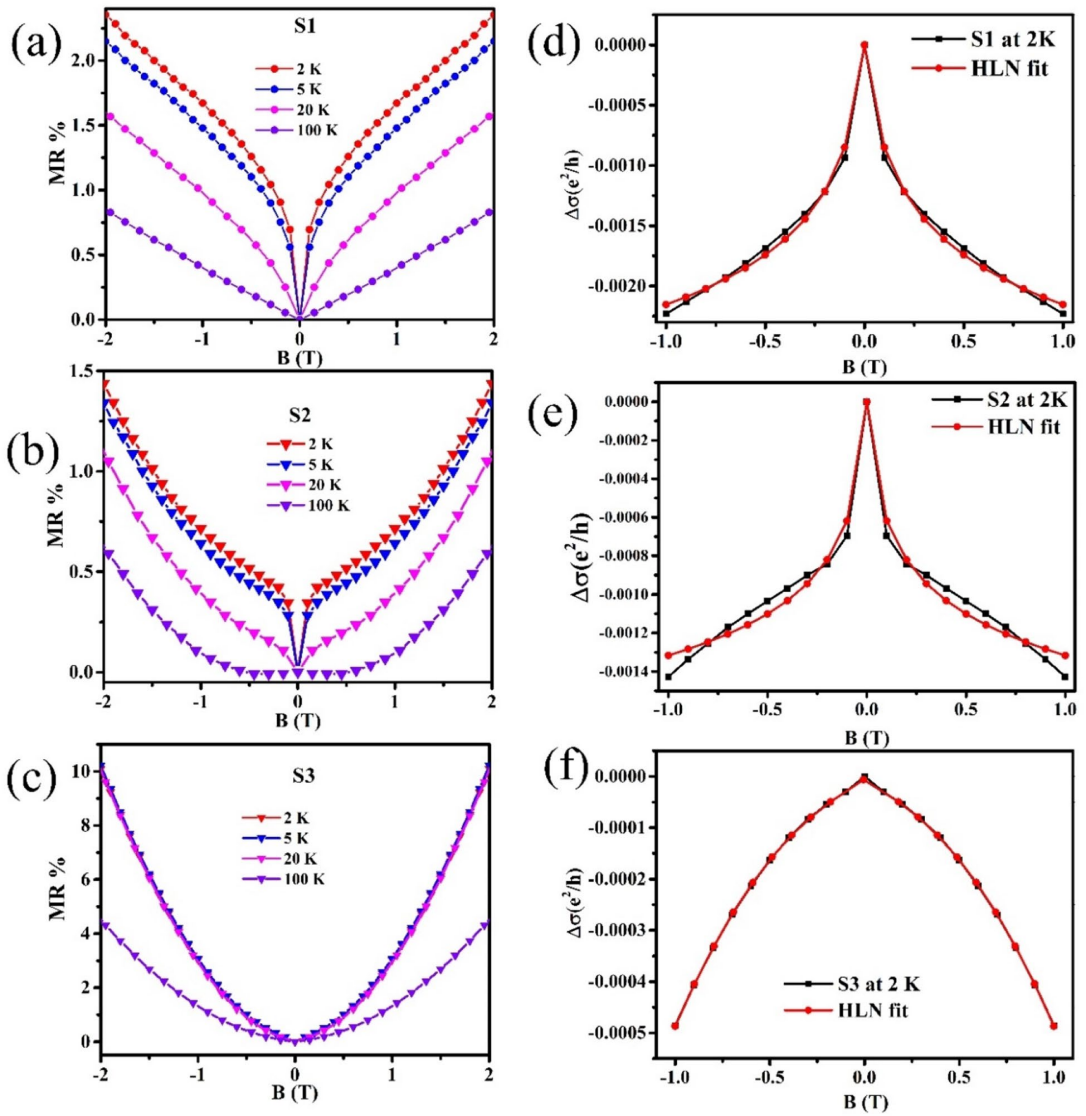
(**a**–**c**) Magneto-resistance (MR) as a function of the perpendicular magnetic field (− 2 to 2 T) at T = 2–100 K for Bi_2_Se_3_ film samples S1, S2 and S3, respectively. (**d, e**) The HLN fitted magneto-conductance data for samples S1 and S2, respectively. (**f**) The modified HLN fitted magneto-conductance curve for thicker sample S3.

Figure 6a–c represents the magnetoresistance (MR) versus applied magnetic field plot of samples S1, S2 and S3, respectively at temperatures 2–100 K by employing the formula of magnetoresistance^14^:

1 $$MR\% = \frac{R\left( B \right) - R\left( 0 \right)}{{R\left( 0 \right)}} \times 100$$

where R (B) and R (0) are resistances of film at applied field and zero magnetic field, respectively.

Temperature dependent MR data of S1 in Fig. 6a shows that in a perpendicular low magnetic field of − 2 to 2 T and at temperature of 2 K and 5 K, MR forms a sharp cusp like characteristic around B = 0 T. This is a quantum interference phenomenon known as WAL and is thought to be responsible for this cusp. But, at higher temperatures, at 20 K this WAL signature reduces and vanishes at 100 K and this cusp takes a parabolic shape which is opposite to WAL^14^. For sample S2, MR is shown in Fig. 6b and it clearly showed that the WAL effect in S2 is sustained for temperatures at 5 K. It has been observed that with increasing temperature, sharp cusps become weaker and the cusp shape starts to broaden after 5 K and a nearly parabolic shape of MR occurs at 20 K. For sample S3, even at 2 K, there is no proper signature of WAL effect and an MR cusp has parabolic shape at 2–100 K. Also, MR of film overlapped, which suggests that for sample S3, temperature does not play a vital role and WAL diminishes even at low temperatures. Rather than showing a sign of WAL, the MR data of S3 exhibits a classical LMR effect which is due to the dominance of bulk states over surface states in total conductivity and can only be found in less resistive samples^11^. Although all films show a positive MR in a lower magnetic field but only samples S1 and S2 showed clear WAL cusp and their magnetoconductivity data can be fitted using HLN theory^34^. The WAL effect in a topological system is traditionally fitted by HLN theory^34^

2 $$\Delta \sigma \left( B \right) = - \frac{{\alpha e^{2} }}{{2\pi^{2} }}\left[ {\ln \left( {\frac{{B_{\Phi } }}{B}} \right) - \Psi \left( {\frac{1}{2} + \frac{{B_{\Phi } }}{B}} \right)} \right]$$

The above Eq. (2) is 2D model of HLN theory, where **α** is a coefficient that indicates the total number of independent conducting channels present in the film, ${\varvec{B}}_{{\varvec{\varPhi}}} = \frac{\hbar}{{4{\varvec{eL}}_{{\varvec{\varPhi}}}^{2} }}$ is the characteristic field and here L_Φ_ is the effective dephasing length and **Ψ** digamma function. The value of α vary from 0.16 to 1.5 and in some case α found to be as large as 3 for TI in several experimental reports^8,35^.

Figure 6d, e represents the low field magnetoconductivity data from − 1 to + 1 T at temperature 2 K for samples S1 and S2, respectively. Here we have fitted the magnetoconductivity curves using HLN equation. The obtained α values for sample S1 and S2 are found to be 0.65 and 0.83, respectively at temperature 2 K which is increasing with film thickness. The increasing value of α with increasing film thickness describes that the contribution of surface states to the conduction is weak for thicker film^8^. The S1 and S2 samples magnetoconductivity curves have a clear WAL cusp which confirms the less elastic scattering and spin orbit scattering in low magnetic field region at 2 K^23^.

For thicker S3 sample, the MR data clearly shows the less WAL effect and LMR approximation was introduced by which we can fit the parabolic shape of magnetoconductance data for sample S3. The modified HLN equation can be written as^36^:

3 $$\Delta \sigma \left( B \right) = - \frac{{\alpha e^{2} }}{{2\pi^{2}\hbar }}\left[ {\ln \left( {\frac{{B_{\Phi } }}{B}} \right) - \Psi \left( {\frac{1}{2} + \frac{{B_{\Phi } }}{B}} \right)} \right] + \lambda B$$

where the first term is HLN equation and the second term **λ** is a linear term which is taken into the account to reduce the effect of classical linear MR^36^. After applying the modified HLN equation, the α value was found to be 1.56 for sample S3 (Fig. 6f). Gopal et al. reported an α value of 0.7 for a metallic film of Bi_2_Se_2_Te ^11^. Also, an α value in range of 0.8 to 2.9 was reported for different metallic thin films of Bi_2_Te_3_^37^. Further, Zhang et. al. reported that the value of α varied from − 0.34 to − 0.63 with varying thickness of Bi_2_Se_3_ films from 30 to 150 nm ^21^. In our case, the extracted value of α for S1 and S2 samples falls in range of 0.5 to 1 which represents that insulating bulk and surface robustness in prepared sputtered Bi_2_Se_3_ thin films. However, the value of α for sample S3 is obtained to be 1.56 which suggests that the conduction in sample S3 is most likely governed by the bulk state.

## Summary

In summary, we have deposited high crystalline and c-axis oriented Bi_2_Se_3_ thin films on sapphire (0001) with different thicknesses using magnetron sputtering system. Raman spectra show the good structural quality of deposited films and the AFM study confirms the formation of the low surface roughness Bi_2_Se_3_ thin films. The high purity of deposited films was confirmed by chemical and electronic states analyzed using XPS. Further, devices were made using Bi_2_Se_3_/sapphire (0001) thin films to study the low-temperature transport measurements. The resistivity vs temperature measurements show the metallic behavior of Bi_2_Se_3_ thin films and the positive magneto-resistance value observed which confirms the gapless topological surface states in these samples. Further, it was found that the number of transport channel (α) increase from 0.65 to 0.83 with increasing the thickness of films from 40 to 80 nm as both top and bottom surface states contribute to the conductivity of Bi_2_Se_3_ thin films. Thicker film (160 nm) showed the α value of 1.56 and conductance is likely governed by bulk state. Our studies revealed the capability of magnetron sputtering system to grow good quality Bi_2_Se_3_ thin films comparable to MBE and showed the change in the WAL phenomena with thickness which suggest the futuristics applications of large area sputtered films for quantum-based devices.

## Experimental details

Bi_2_Se_3_ thin films of different thicknesses were deposited on sapphire (0001) substrate by radio frequency (RF) magnetron sputtering system (base pressure: < 2 × 10^–7^ mbar). The magnetron sputtering having two vacuum chambers: load lock chamber and main growth chamber. The substrates were cleaned using acetone, iso-propyl alcohol and de-ionized water followed by  drying with argon gas before loading in load lock chamber. The substrates were transferred from load lock chamber to the main chamber without exposing growth chamber to the environment. A commercially available Bi_2_Se_3_ sputtering target (Purity 99.99%) was employed for deposition. The deposition process was carried out in an ultra-pure argon atmosphere (Purity 99.9999%). During deposition, the substrate temperature was fixed at 425 °C and Ar plasma was achieved at RF power of 10 W. Deposited films were then post-selenized in a tubular furnace at an operating temperature of 300 °C to maintain the stoichiometry. Three Bi_2_Se_3_ thin films having thicknesses of 40, 80 and 160 nm, referred as samples S1, S2 and S3, respectively. The deposition rate and thickness of Bi_2_Se_3_ thin films were estimated using AFM and cross-sectional SEM images. For device fabrication, shadow mask was used to deposit Cr/Au contacts having thickness ~ 5 nm/ ~ 75 nm by thermal evaporator.

The HR-XRD was used to study the crystalline nature and phase of deposited thin films using a CuK_α1_ x-ray source (λ = 0.15406 nm). Raman spectroscopy was used to examine the structural properties with an Ar + laser source (λ ~ 514.5 nm) in the back scattering geometry. Surface morphology and surface roughness of Bi_2_Se_3_ thin films was investigated by AFM in tapping mode. To determine the chemical and electronic nature of deposited Bi_2_Se_3_ thin films, XPS technique was employed. Low temperature transport measurements were carried out in a Quantum design physical properties measurement system (PPMS) and standard four probe geometry was used for magneto-transport measurements. All measurements performed under the magnetic field of − 12 T to 12 T up to the 2 K temperature.

## Data Availability

All data generated or analysed during this study are included in this published article.
